# A new species of inseminating catfish of the genus *Tympanopleura* (Siluriformes: Auchenipteridae) from the Ituxi River, Amazon River basin, northern Brazil, revealed by integrative taxonomy

**DOI:** 10.1111/jfb.70445

**Published:** 2026-04-13

**Authors:** Frank Raynner V. Ribeiro, Cárlison Silva‐Oliveira, Valdenor Magalhães, Lucas Gama, Lúcia H. Rapp Py‐Daniel

**Affiliations:** ^1^ Instituto de Ciências e Tecnologia das Águas Universidade Federal do Oeste do Pará Santarém Pará Brazil; ^2^ Campus Itaituba Instituto Federal de Educação, Ciência e Tecnologia do Pará Itaituba Pará Brazil; ^3^ Programa de Pós‐Graduação em Biologia de Água Doce e Pesca Interior Instituto Nacional de Pesquisas da Amazônia Manaus Amazonas Brazil; ^4^ Coleção de Peixes, Programa de Coleções e Acervos Instituto Nacional de Pesquisas da Amazônia Manaus Amazonas Brazil

**Keywords:** Amazon fish, biodiversity, conservation status, systematics, taxonomy

## Abstract

A new species of the genus *Tympanopleura* is described from the Iquiri River, a tributary of the Ituxi River, a right‐bank tributary to the Purus River, Amazon River, northern Brazil. The new species is distinguished from all its congeners by a combination of features, such as the presence of an intensely pigmented square‐shaped blotch on the supraoccipital, a semicircular dark blotch above each eye and an inconspicuous vertical bar on the caudal‐fin base (except *Tympanopleura piperata*), and by a combination of meristic and morphometric character states. Preliminary molecular analyses demonstrate genetic distance values of 2.4% of cytochrome oxidase, subunit I, divergence between the new species and *T*. *piperata* and 8.0%–13.0% between the new species and the other congeners. The new species shows several reproductive adaptations to copulation and internal insemination. Considering the period during which prenuptial and nuptial specimens were available, we can presume that its reproductive period occurs when the water level increases. In addition, an identification key for the species of *Tympanopleura* is provided.

## 1 INTRODUCTION

*Tympanopleura* Eigenmann, 1912, was proposed to accommodate *Tympanopleura piperata* Eigenmann, 1912, a new species described based on six specimens from the Essequibo River, Guyana. The genus was originally distinguished from the remaining Auchenipteridae mainly by the possession of an enlarged gas bladder and sides of the coelomic cavity with a pseudotympanum. However, *Tympanopleura* has been recognized historically as a junior synonym of *Ageneiosus* Lacépède, 1803, based on the argument that the diagnostic characters proposed by Eigenmann (1912) can vary largely intraspecifically and ontogenetically (e.g. Birindelli, 2014; Britski, 1972; Ferraris Jr. & Vari, 1999; Ferraris Jr., 1988; Royero, 1999; Walsh, 1990).

*Tympanopleura* was taxonomically revised by Walsh et al. (2015), who recognized its valid generic status, demonstrating that all included species, usually referred to as *Ageneiosus*, share a large and cordiform non‐encapsulated gas bladder; a prominent pseudotympanum, readily visible externally; and, except by *T*. *piperata*, a gas bladder with a pair of posterior terminal diverticula. Furthermore, based on a combined morphological and molecular analysis, Calegari et al. (2019) recovered *Tympanopleura* as a monophyletic group, diagnosed by six molecular and three non‐exclusive morphological synapomorphies: eye very large, occupying almost entire head depth; upper gill rakers conical; and postzygapophysis of compound centrum extended to the seventh vertebra. Hashimoto et al. (2020) also recovered *Tympanopleura* as monophyletic using cytochrome oxidase, subunit I (COI) data, although their study did not provide a revised definition or diagnosis for the genus.

Currently, the genus includes six valid species: *Tympanopleura atronasus* (Eigenmann & Eigenmann, 1888), *Tympanopleura brevis* (Steindachner, 1881), *Tympanopleura cryptica* Walsh et al., 2015, *Tympanopleura longipinna* Walsh et al., 2015, *T*. *piperata* and *Tympanopleura rondoni* (Miranda Ribeiro, 1914). Except for *T*. *piperata*, all *Tympanopleura* species are distributed only in the Amazon River basin (Fricke et al., 2025; Walsh et al., 2015).

Here we describe a new species of *Tympanopleura* collected during recent expeditions in the Iquiri River, a tributary of the Ituxi River, a right‐bank tributary to the Purus River, Amazon River basin, northern, Brazil.

## 2 MATERIALS AND METHODS

### 2.1 Ethics statement

Specimens of the new species were collected in compliance with Brazilian laws, through licence number 11561‐1 granted by Instituto Chico Mendes de Conservação da Biodiversidade (ICMBio). No experimental procedures were performed with live fish. After collection, specimens were euthanized by immersion in 600 mg L^−1^ eugenol solution (clove oil) (Fernandes et al., 2017). Samples for molecular analyses were fixed in 96% ethanol in the field and stored in freezers in the laboratory. All specimens were fixed in 10% formalin and subsequently transferred to 70% ethanol after 3 days.

### 2.2 Morphological analyses

Counts and measurements were made on the left side of the specimens whenever possible. Standard length (*L*_S_) is expressed in millimetres, and other body measurements are expressed as a percentage of *L*_S_, except those that represent subunits of the head, which are given as percentages of head length (*L*_H_). Body measurements were taken as point‐to‐point linear distances using digital callipers and recorded to 0.01 mm, according to Ribeiro and Rapp Py‐Daniel (2010). Counts of fin rays and gill rakers were obtained from alcohol‐preserved, cleared and stained specimens.

Fin‐ray counts include the anteriormost spine (capital Roman numeral) or unbranched ray (lower‐case Roman numeral), and all subsequent branched rays (Arabic numeral). The two posteriormost dorsal‐ and anal‐fin rays articulating with the last pterygiophore of each fin were counted as separate rays. Caudal‐fin ray counts included all principal rays (i.e. all inner branched rays and the first unbranched ray of the dorsal and ventral lobes, also referred to as ‘outer principal rays’). Gill rakers of the anterolateral row were counted on the first branchial arch. Any gill raker located at the articulation between the epibranchial and ceratobranchial was included in the epibranchial count.

Osteological preparations were cleared and counterstained (c&s) for cartilage and bone using the method of Taylor and van Dyke (1985). Osteological terminology follows Lundberg and Baskin (1969) and Arratia (2003). Vertebral, pleural rib and procurrent caudal‐fin ray counts were taken from c&s and x‐rayed specimens. Vertebral counts follow Vari et al. (2013), including the five elements incorporated into the Weberian complex and elements of the hypural complex as a single element. Images of structural details were captured using a digital camera coupled to a stereomicroscope.

Institutional abbreviations follow Sabaj (2020). In the list of comparative material examined, the museum abbreviation and catalogue number are followed by the total number of specimens in the lot, the range of the standard length, an indication of c&s specimens and abbreviated collection data.

### 2.3 Molecular analyses

Genetic samples were processed at the Animal Evolution and Genetics Laboratory of the Federal University of Amazonas (UFAM). DNA extraction followed the phenol–chloroform method (Sambrook & Russell, 2001). The DNA obtained from each sample was quantified using the Nanodrop 2000 device and visualized on 1% agarose gel. The amplified fragment was the mitochondrial gene COI with ~680 base pairs.

Gene amplification and sequencing followed the protocol and primers COIFishF2 (5′‐CGACTAATCATAAAGATATCGGCAC‐3′) and COIFishR1 (5′‐TTCAGGGTGACCGAAGAATCAGAA‐3′) proposed by Colatreli et al. (2012). Polymerase chain reaction (PCR) was performed in a total volume of 15 μL containing 6.7 μL of ddH_2_O, 1.2 μL of MgCl_2_ (25 nM), 1.2 μL of deoxyribonucleotide triphosphate (dNTP) (10 mM), 1.5 μL of 10× PCR buffer (100 mM Tris–HCl, 500 mM KCl), 1.2 μL of Primer COIFishF2, 1.2 μL of Primer COIFishR1, 0.5 μL of bovine serum albumin (BSA), 0.5 μL of Taq DNA polymerase and 1 μL of DNA. The conditions for PCR were as follows: 1 cycle of denaturation at 93°C for 60 s, 35 cycles of annealing at 50°C for 45 s, extension at 72°C for 60 s and final extension at 72°C for 10 min. Sample purification was carried out using PEG 8000 (polyethylene glycol) and alcohol (Paithankar & Prasad, 1991), and sequence reactions were carried out using the BigDye Terminator version 3.1 Cycle Sequencing Kit (Life Technologies). Fragment sequencing was performed on an ABI 3130XL sequencer.

For molecular analyses sequences deposited in GenBank (see accession numbers) were used for the other congeneric species. For this study, we generated two sequences of the new species and submitted them to GenBank (accession numbers: PV261062, PV261063). The COI sequences of *T. atronasus*, *T. brevis*, *T. longipinna*, *T. piperata*, *T. rondoni* and *Tympanopleura* sp. were obtained from GenBank, and their accession numbers are provided in Appendix.

The obtained sequences were edited in BioEdit (Hall, 1999) and aligned in MEGA X (Kumar et al., 2018) using the CLUSTAL‐W algorithm (Thompson et al., 1994). To calculate the genetic distance between species, we used the Kimura 2‐parameter (K2P) model in which we assumed the maximum intraspecific distance to be 2%, which is the COI threshold more commonly used in studies with Neotropical fishes (Jacobina et al., 2018).

To understand the relationships between groups, we estimated a maximum likelihood tree, conducted using the General Time Reversible + G model, with a bootstrap support of 1000 replicates performed in MEGA 11 (Tamura et al., 2021).

Two unilocus species delimitation analyses were performed with the aim of defining the molecular operational taxonomic units (MOTU), one based on molecular distance [assemble species by automatic partitioning (ASAP)] and the other based on coalescence [Bayesian Poisson tree process (bPTP)]. ASAP analysis was performed through the interface available online (https://bioinfo.mnhn.fr/abi/public/asap/asapweb.html), using the K2P model. For bPTP analysis, a Bayesian inference tree was generated, with 20,000 generations, using MrBayes software, version 3.2 (Ronquist et al., 2012), and the analysis was performed through the online interface, https://species.h-its.org/ptp/.

## 3 RESULTS

### 3.1 *Tympanopleura personata*, new species

**FIGURE 1:**
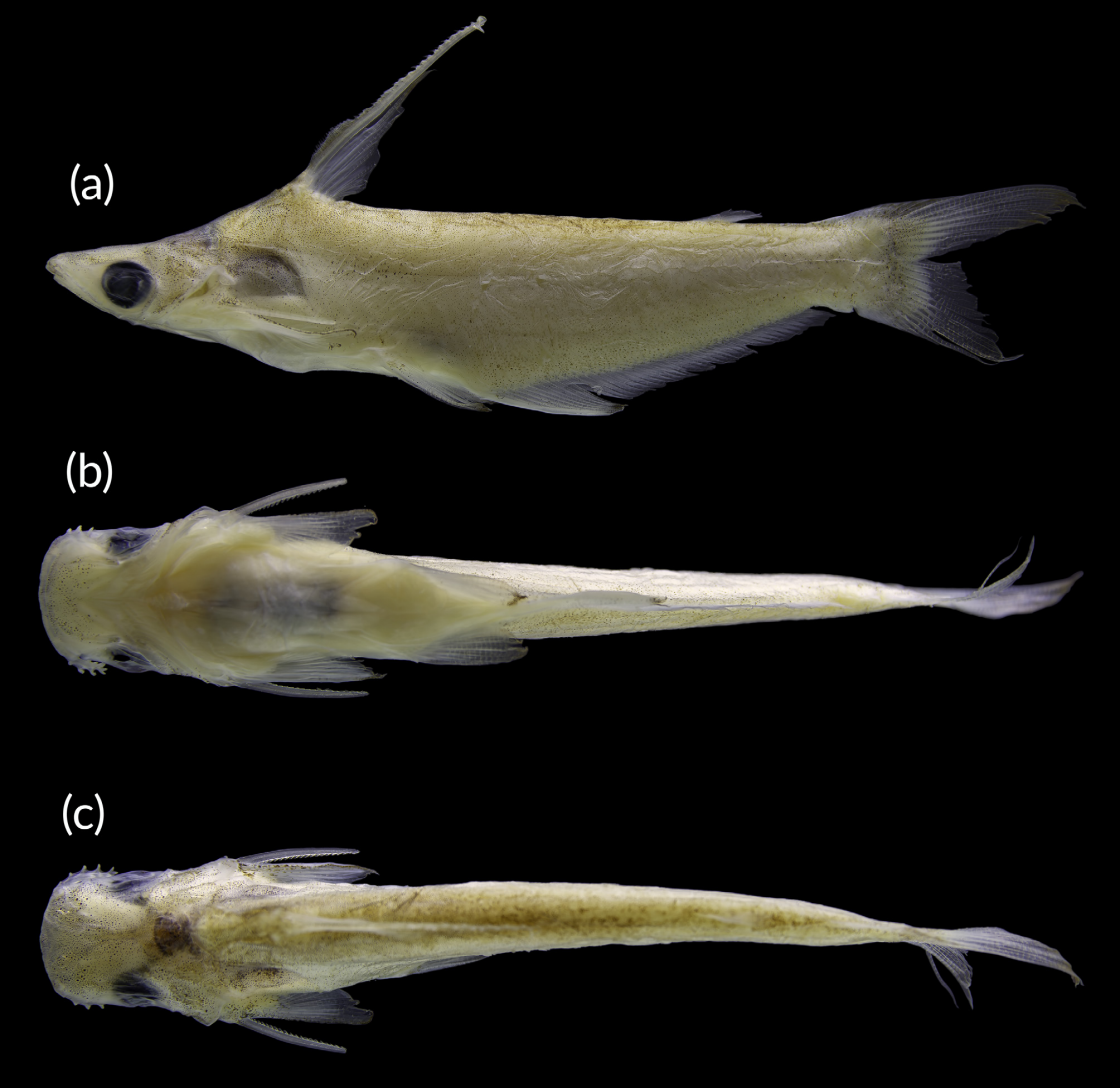
*Tympanopleura personata* in (a) lateral, (b) ventral and (c) dorsal views, UFOPA‐I 1373, 77.8 mm *L*_S_, holotype, male, Iquiri River, Lábrea, Amazonas, Brazil.

**FIGURE 2:**
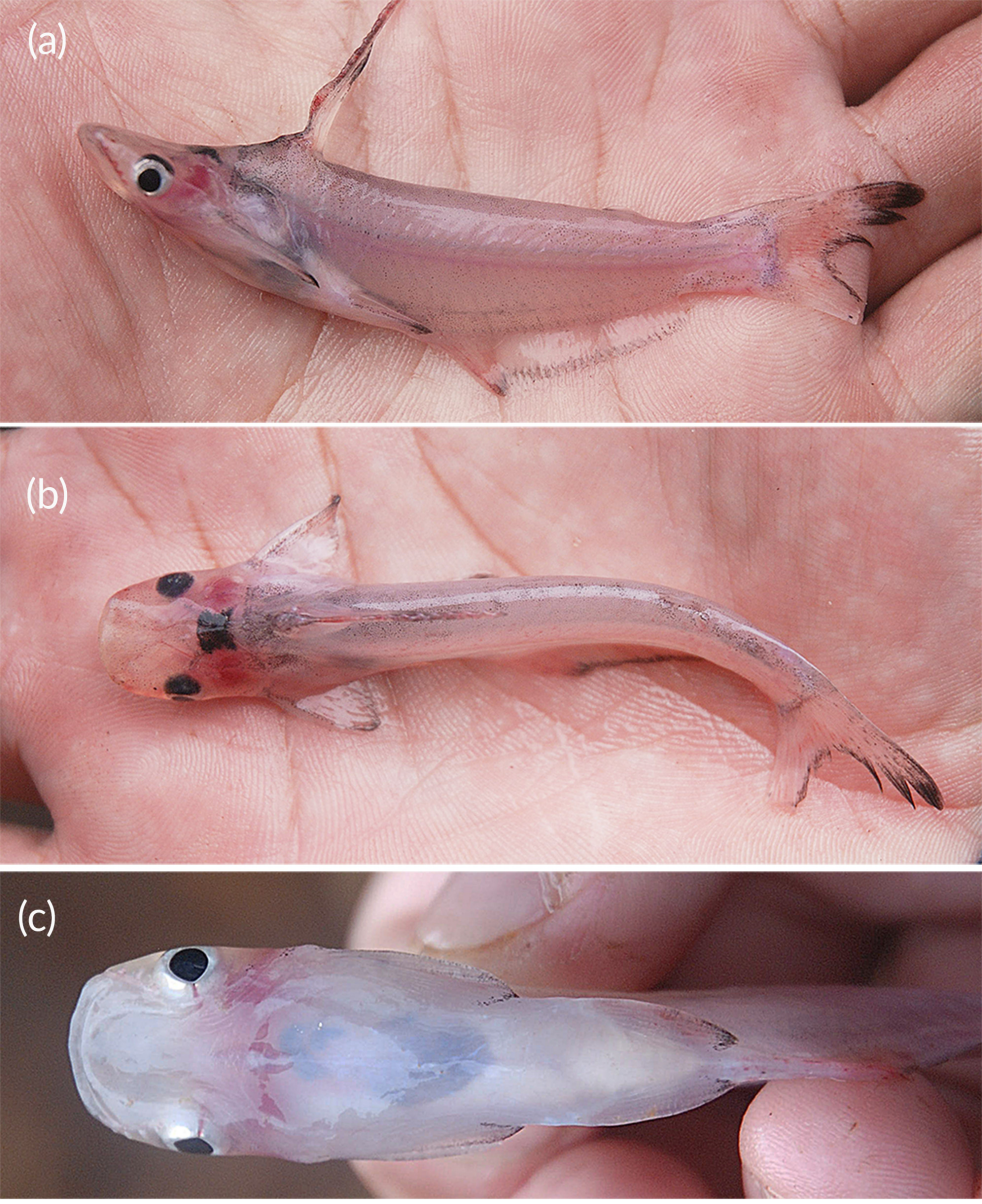
*Tympanopleura personata* in life, in dorsal (a) lateral, (b) ventral views and (c) dorsal views, UFOPA‐I 1374, 84.0, mm *L*_S_, paratype, Iquiri River, Lábrea, Amazonas, Brazil.

Figures 1 and 2; Table 1

**TABLE 1:** Morphometric data of *Tympanopleura personata*, new species.

Measurements	HT	Low	High	Mean	SD	*n*
Standard length (mm)	77.8	64.8	85.7	74.5	7.6	15
Percentage of standard length						
Predorsal length	29.0	27.2	29.3	28.3	0.6	15
Pre‐anal length	53.2	51.8	54.1	53.0	0.8	15
Prepelvic length	41.1	40.9	43.4	42.0	0.7	15
Head length	23.8	22.4	24.6	23.4	0.6	15
Caudal peduncle length	11.8	11.1	13.8	12.2	0.8	15
Caudal peduncle depth	9.5	8.5	9.8	9.2	0.4	15
Dorsal‐fin end to adipose‐fin origin	41.9	41.9	45.6	44.0	1.1	15
Anal‐fin base	40.1	34.3	40.5	38.4	1.5	15
Pectoral‐fin length	12.9	10.7	12.9	12.0	0.5	15
Dorsal‐fin length	20.2	14.1	20.6	18.0	2.0	15
Dorsal spine length	30.9	12.8	32.1	27.6	5.5	10
Pectoral spine length	15.5	14.3	15.7	15.1	0.5	13
Body depth	19.6	17.7	20.0	19.0	0.6	15
Body width	14.2	12.5	14.8	13.4	0.7	15
Percentage of head length						
Head depth	58.3	54.7	60.2	57.7	1.6	15
Head width	73.5	66.8	75.5	72.0	2.9	15
Interorbital width	61.6	55.8	63.9	60.1	2.6	15
Snout length	41.4	41.4	50.6	46.9	2.5	15
Snout projection	5.4	3.2	6.7	4.8	0.8	15
Internarial length	18.9	17.2	21.0	19.0	1.1	15
Anterior internarial width	31.9	28.8	32.3	30.5	1.2	15
Posterior internarial width	23.8	21.4	26.9	24.3	1.6	15
Horizontal eye diameter	27.0	25.6	31.8	28.0	1.6	15
Mouth width	54.6	47.9	55.0	51.2	2.2	15
Second nuchal plate width	25.1	24.8	30.3	26.9	1.6	15
Second nuchal plate length	38.9	34.5	39.6	36.6	1.9	15

*Note:* Range includes the holotype (HT).

Abbreviations: *n*, number of specimens; SD, standard deviation.

urna:lsid:zoobank.org:pub:BE0BABC5‐DF6A‐441D‐B977‐F89140917D65

urna:lsid:zoobank.org:act:7E4F78A0‐63DB‐4A01‐8A20‐0FE9FBAFB60D

#### 3.1.1 Holotype

UFOPA‐I 1373, 77.8 mm *L*_S_, male, Brazil, Amazonas, Lábrea, Iquiri River, tributary of Ituxi River, Purus River, Amazon River basin, 8°59′42.60″, S 66°21′22.04″ W, 9 February 2022, Cárlison Silva‐Oliveira, M. Lima and Valdenor Magalhães.

#### 3.1.2 Paratypes

All collected with the holotype: UFOPA‐I 1374, 11 (1 c&s), 69.2–85.7 mm *L*_S_ (9 male and 2 presumed female). INPA 61048, 5 (2 x‐rayed), 64.8–69.4 mm *L*_S_ (4 male and 1 presumed female).

### 3.2 Diagnosis

*Tympanopleura personata* is distinguished from all congeners by having a unique colouration of the head, consisting of the combination of an intensely pigmented square‐shaped blotch on the supraoccipital and a semicircular dark blotch above each eye (Figure 2; Walsh et al., 2015: Figure 1e). It also differs from all congeners, except *T*. *piperata*, by the caudal‐fin pigmentation, consisting of a patch of scattered melanophores forming an inconspicuous vertical bar on the caudal‐fin base, most visible in live or freshly preserved specimens (Figure 2). *Tympanopleura personata* differs from *T*. *piperata* by having a longer caudal peduncle (11.1%–13.8% *L*_S_ vs. 7.6%–10.5% *L*_S_) and a smaller distance between the insertion of the last dorsal‐fin ray and adipose‐fin origin (41.9%–45.6% *L*_S_ vs. 45.9%–55.4% *L*_S_). Additionally, *T*. *personata* can be distinguished from *T. atronasus*, *T. cryptica*, *T. brevis*, *T. piperata* and *T. rondoni* by having more anal‐fin rays (36–40, mode 39 vs. 23–30, mode 27 in *T. atronasus*; 23–30, mode 29 in *T. cryptica*; 31–36, mode 33 in *T. brevis*; 31–38, mode 35 in *T. piperata*; and 28–37, mode 31 in *T. rondoni*) and few total gill rakers (17–20, mode 19 vs. 20–24, mode 23 in *T*. *brevis*; 21–26, mode 22 in *T*. *cryptica*; 19–25, mode 23 in *T*. *longipinna*; and 24–33, mode 29–30 in *T*. *rondoni*).

### 3.3 Description

Morphometric data are presented in Table 1. Body elongate, depth of trunk proportionally greater than its width. Dorsal profile concave from snout tip to dorsal‐fin origin; straight to slightly convex from behind dorsal fin to end of body. Isthmus and abdomen flat or slightly convex to pelvic‐fin origin. Ventral profile of body convex from lower lip to anal‐fin origin; straight, posterodorsally inclined along anal‐fin base. Dorsal and ventral profiles of caudal peduncle concave. Body moderately compressed at pectoral‐fin insertion, strongly compressed posteriorly to pelvic‐fin base.

Head wide, depressed anteriorly, progressively elevated posteriorly; slightly longer than wide; dorsal profile concave to dorsal‐fin origin. Mouth wide and subterminal, upper jaw projecting slightly beyond lower jaw by distance less than horizontal eye diameter. Mouth corner roughly in line with posterior nare. Snout relatively long, about half head length, anterior margin rounded.

Premaxillary and dentary tooth patches relatively narrow, teeth minute, conical to slightly curved; in irregular rows of five to six rows medially on premaxilla, tapering to two to three rows at sides; five to six rows medially on dentary, increasing to seven to eight rows at sides. Eye moderate in size (24.2%–30.2% *L*_H_), covered by relatively thin layer of epidermis; eyes lateral, equally visible from dorsal and ventral views (Figure 1). Cranial fontanel elongate, open from mesethmoid to a plane approximately in line with midpoint of orbit. Anterior internarial width greater than posterior. Anterior nares slightly lateral to distal tip of mesethmoid wings and directed forward; posterior nares not bordering lateral margin of frontal, with a low epidermal flap around margin, slightly raised posteriorly. Branchiostegal membrane broadly attached to isthmus, behind plane through rear margin of orbit; supported by seven branchiostegal rays. Total gill rakers on anterolateral margin of first arch 17–20 (mode 19, *n* = 16); epibranchial with 5–7 (mode 6), ceratobranchial with 12–13 (mode 13). Longest gill rakers near middle of arch thin and crenulate on medial margin; tapering, short, pointed near ends of each arch. Maxillary barbels of females or non‐nuptial males minute, filiform, concealed in premaxillary groove.

Dorsal‐fin rays II,6, consisting of small spinelet, spine, branched rays; first branched ray longest, subsequent rays decreasing gradually in length; tip extending posterior to vertical through pelvic‐fin origin when fin adpressed. Dorsal‐fin origin located posterior to vertical through pectoral‐fin origin. Dorsal‐fin spine relatively thin, weak in females or non‐nuptial males, depressed spine reaching about one third or less the distance between dorsal‐fin origin and adipose‐fin origin. Anterior margin of dorsal‐fin spine weakly to moderately crenulated with single row of low‐crowned denticulations, more spaced distally. Lateral margins of spine nearly smooth. Posterior margin of dorsal‐fin spine with 12–19 relatively small, conical, retrorse serrae. Adipose fin small, forming free lobe posteriorly; its origin anterior to posterior limit of anal‐fin base. Pectoral fin I,9–10 (mode = 10, *n* = 17); first branched ray longest, subsequent rays decreasing gradually in length. Pectoral‐fin spine well developed, rigid, distally tapering, sharp; anterior, dorsal and ventral surfaces smooth. Posterior margin of spine with single series of 18–21 uniformly spaced, unicuspid, retrorse serrae along entire length. Postcleithral process absent. Pelvic‐fin rays i,6; first branched ray longest, subsequent rays progressively shorter. Pelvic‐fin origin slightly posterior to tips of longest adpressed pectoral‐fin rays; distal margin of fin straight to slightly convex. Anal fin long, with 36–40 rays (mode 39, *n* = 17), distal margin concave. First five anal‐fin rays of prenuptial and nuptial males unbranched, elongated and thickened to form intromittent organ; gonopore located distally on leading edge of anal fin about one third to one‐half distance from base to tip of longest ray.

Caudal fin forked, with 8 + 9 principal rays; 15–17 (3) upper and 13–14 (3) lower procurrent rays. 16 (3). Caudal vertebrae 29 (2) or 30 (1). Total vertebrae 41 (2) or 42 (1). Pleural rib pairs 5 (3). Pseudotympanum large, triangular, opaque to semi‐translucent (Figure 1).

### 3.4 Sexual dimorphism

Observed in 13 nuptial and prenuptial males. Predorsal profile slightly more angled upwards than in females, juveniles or non‐nuptial males. Maxillary barbel of nuptial males entirely ossified, rigid, thickened. Barbel elongate, reaching beyond the anterior eye margin when adpressed; curved inwards towards side of head. Two rows of sharp recurved hooks on anterolateral and posterodistal surfaces of barbel, dorsally oriented. Hooks fully ossified, numbering 5–2 on the anterolateral surface, 1–3 on the posterodistal margin. Articular surface of maxilla expanded for contact with autopalatine. Barbels of prenuptial males thickened, elongated, ossified most of length, with fleshy tip.

Dorsal‐fin spine of prenuptial and nuptial males elongated, rigid, nearly straight (Figure 1). Anterior margin with pungent, unicuspid, antrorse serrae, closely spaced proximally, forming a laminar, ossified protuberance at anterior base of spine; serrae arranged in alternating anterolateral direction on distal half of spine, directed less acutely anterolaterally along middle second to third of spine. Posterior margin with small, irregularly spaced denticulations on proximal half, distal half nearly smooth. Dorsal‐fin spine may be hyperextended anteriorly to ~45° dorso‐anterior angle. First branched dorsal‐fin ray longer than in nonbreeding males or females.

Unbranched and first few branched anal‐fin rays thickened and elongated to form a tubular intromittent organ bound by integument to the anterior margin of the anal fin. Urogenital opening of nuptial male displaced as a simple pore at the distal tip of intromittent organ.

### 3.5 Colour in alcohol

Overall ground colour of body light tan to cream. Top of head with intensely pigmented square‐shaped blotch on the supraoccipital. Dark brown, semicircular blotch above each eye (Figure 2). Dorsal region with diffuse concentrations of dark‐brown melanophores extending from nuchal plate to caudal fin, diminishing laterally. Side of body with scattered black melanophores, more darkly pigmented below dorsal fin. An inconspicuous vertical bar at the base of the caudal fin, consisting of fine, scattered black melanophores, sometimes coalescing with dorsum pigmentation. Adipose fin hyaline except for small patch of melanophores extending from midline onto anterior base of fin. Dorsal, pectoral, pelvic, anal and caudal fins with thin bands of black pigment along distal margins. Upper caudal‐fin lobe with dark blotch on distal portion.

### 3.6 Colour in life

Body semi‐translucent. Colour of body similar to those of preserved specimens. Fins more intensely pigmented (Figure 2).

### 3.7 Geographical distribution

**FIGURE 3:**
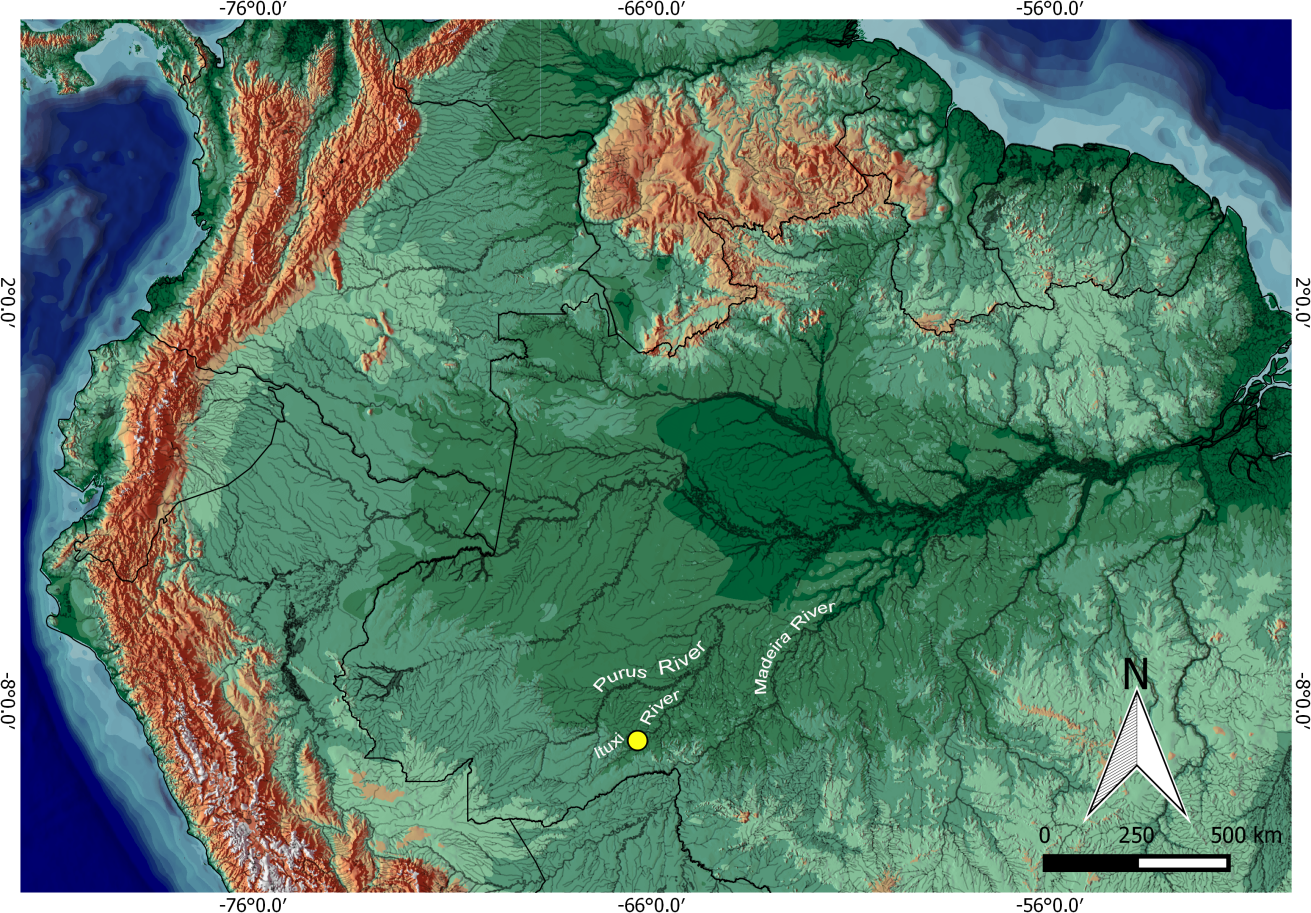
Map indicating the known geographical distribution of *Tympanopleura personata* in the Iquiri River, upper Purus River basin, Amazonas, Brazil.

*T. personata* is known only from its type locality, in the Iquiri River, Ituxi River, a right‐bank tributary of the Purus River, Amazon River basin, northern Brazil (Figure 3).

### 3.8 Conservation status

Despite being known only from the Iquiri River, *T*. *personata* occurs in a well‐preserved area, with no signs of disturbances that could put its known population at risk. Therefore, according to the International Union for Conservation of Nature (IUCN) criteria, *T*. *personata* may be categorized as least concern, though Edgar (2025) argues that such assessments often fail to account for species with limited distribution or cryptic populations, potentially underestimating extinction risks.

### 3.9 Ecological notes

Specimens of *T*. *personata* were collected in a stretch with moderate to fast‐flowing white waters, and substrate consisted predominantly of twigs. Trees and bushes were the main components of the riparian vegetation.

*T. personata* exhibit several reproductive adaptations, many of which are related to copulation and internal insemination, such as anteriormost anal‐fin rays of prenuptial and nuptial males thickened and coalesced to form the intromittent organ of nuptial males; presence of dermal (nuptial) tubercles; and development and ossification of the dorsal‐fin spine, maxillary barbel, and fin rays. *T. personata* were collected in February. Considering the period during which prenuptial and nuptial specimens were available, we can presume that the reproductive period occurs when the water level increases. Some males exhibited peak nuptial barbel and dorsal‐fin dimorphism in February. Based on these specimens, the reproductive period for this species may occur from mid‐to‐late January through February. Reproductive males ranged from 64.8 to 84.0 mm *L*_S_.

### 3.10 Etymology

The specific epithet *personata* is derived from the Latin *personatus*, meaning masked, in reference to the dorsal pigmentation of the head. It is treated as a noun in apposition. Gender is feminine.

### 3.11 Molecular analysis

The final COI dataset comprises six nominal species of *Tympanopleura* (missing *T. cryptica*) and an undescribed species. Genetic distance values showed that the closest species to *T*. *personata* is *T. piperata* with 2.4% of divergence, whereas the genetic distance with other congeners ranged from 8.0% to 13.0% (Table 2).

**TABLE 2:** Genetic distances (%) of *Tympanopleura* species based on mitochondrial COI using Kimura 2‐parameter distance.

	Species	1	2	3	4	5	6	7	8
1	*Tympanopleura personata*								
2	*Tympanopleura piperata*	2.4							
3	*Tympanopleura rondoni*	10.0	8.9						
4	*Tympanopleura atronasus*	10.9	11.6	9.7					
5	*Tympanopleura longipinna*	11.3	10.7	8.2	10.2				
6	*Tympanopleura brevis*	12.2	12.0	12.7	12.0	11.9			
7	*Tympanopleura* sp.	11.6	12.0	10.3	10.5	10.9	11.8		
8	*Ageneiosus inermis*	10.5	10.1	7.9	9.3	9.8	11.4	10.1	

Abbreviation: COI, cytochrome oxidase, subunit I.

**FIGURE 4:**
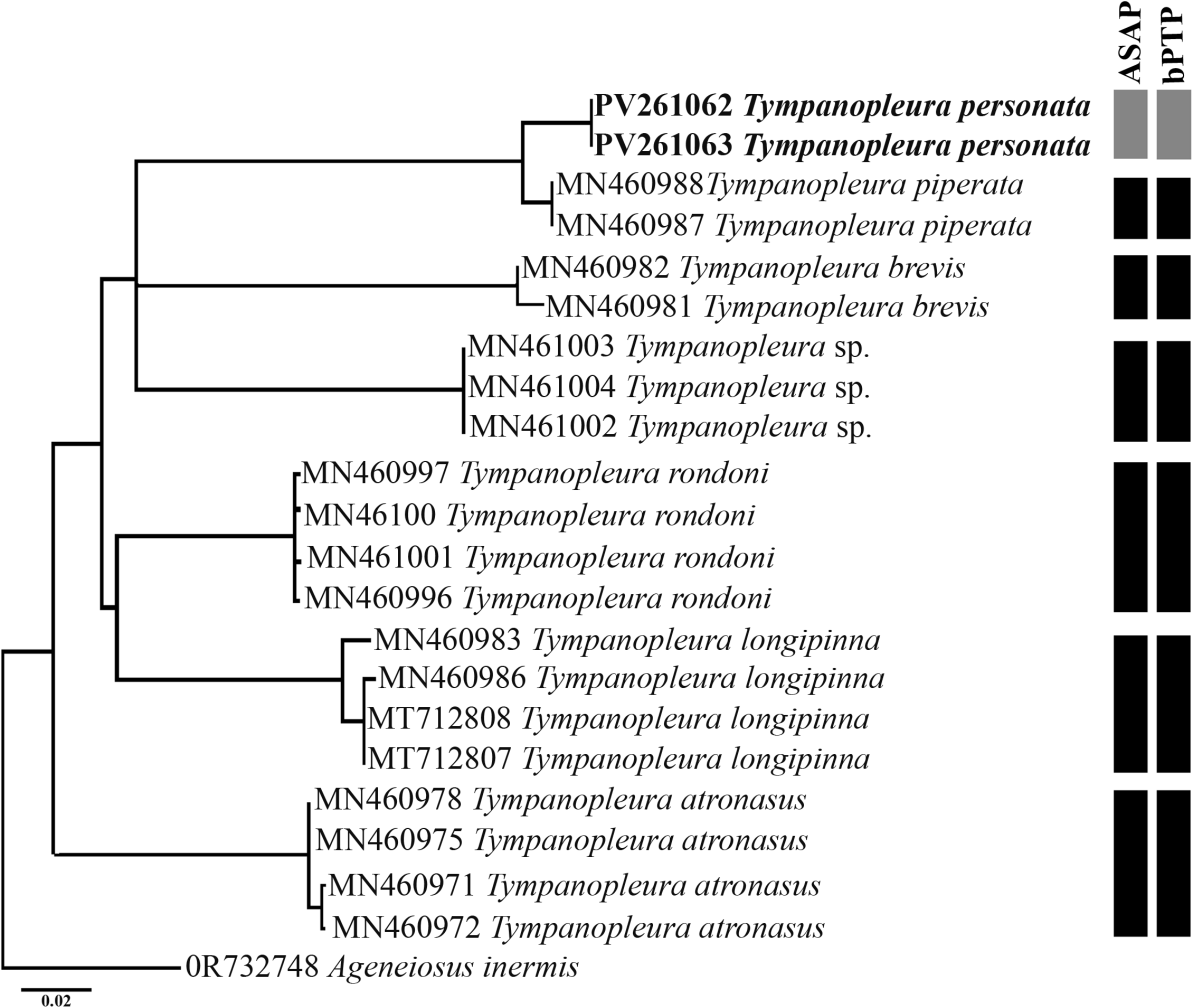
Maximum likelihood tree of *Tympanopleura* species derived from COI (cytochrome oxidase, subunit I) sequences. Values in node branches represent bootstrap support. The columns represent the status of the delimitation of molecular operational taxonomic units by the ASAP (assemble species by automatic partitioning) and bPTP (Bayesian Poisson tree process) methods.

Clusters were consistent across different phylogenetic methods, with high bootstrap support (Figure 4). In two molecular species delimitation methods (ASAP and bPTP) the results were congruent, recovering *T. personata* as a distinct species from the other congeners.

## 4 DISCUSSION

*T. personata* shares all the characters defining the genus (sensu Walsh et al., 2015): presence of a large and cordiform non‐encapsulated gas bladder; a prominent pseudotympanum visible externally; parapophyses of the fourth vertebrae (=Müllerian rami) consisting of large, discoidal plates closely adpressed to the anterodorsal face of the anterior chamber of the gas bladder; and lacking mental barbels in adults. The last character state is shared with *Ageneiosus* species (Ribeiro et al., 2017).

Among *Tympanopleura* species, *T*. *piperata* and *T*. *cryptica* exhibit a condition of a large gas bladder partially ossified, lacking posterior diverticula in *T*. *piperata* (Walsh et al., 2015). The new species shares with *T*. *piperata* and *T*. *cryptica* the condition of a large gas bladder partially ossified and shares with *T*. *piperata* the absence of posterior diverticula. Calegari et al. (2019) reported the condition of an unossified gas bladder in *Ageneiosus lineatus* Ribeiro, Rapp Py‐Daniel & Walsh, 2017, with implication in the genus diagnosis of *Ageneiosus*. Examination of the specimens of *A. lineatus* that served as the basis of the study of Ribeiro et al. (2017) (INPA 35931, INPA 33975, UFOPA‐I 665) has shown that the species possess an ossified gas bladder, as stated by the authors. The gas bladder of *T*. *cryptica*. *T*. *personata* and *T*. *piperata* is only partially ossified (Walsh et al., 2015), whereas *A*. *lineatus* has a more ossified and reduced gas bladder. Moreover, Ribeiro et al. (2017) distinguished *Ageneiosus* from *Tympanopleura* by a combined presence of reduced and completely ossified gas bladder in adults, except in *Ageneiosus pardalis* Lütken, 1874.

Specimens of *T*. *personata* may easily be confused with those of *T. piperata*, as they share an overall morphological appearance and a significantly similar condition of caudal‐fin pigmentation, unique among its congeners. However, both the morphological and molecular comparisons between the specimens of *T*. *personata* and *T. piperata* suggest that these are distinct species. In general, genetic distances between *Tympanopleura* species are high, with more than 10% in most cases (Hashimoto et al., 2020). *T. personata* and *T. piperata* presented the smallest genetic distance among all congener*s* (2.4%). Although the two species are similar in overall appearance, they can be distinguished by the pigmentation of the dorsal region of the head and by meristic and morphometric characters (see Section 3.2). Furthermore, apparently, they are not sympatric species, lacking records of specimens collected together.

In addition to morphological diagnosis and COI genetic distances, the species delimitation analyses performed using ASAP and bPTP provided congruent results in supporting *T*. *personata* as a distinct evolutionary lineage. Both methods consistently recovered two independent MOTUs, one corresponding to *T*. *personata* and the other to *T*. *piperata*. This pattern is visually evident in Figure 4, where *T. personata* forms a well‐delimited and exclusive clade, receiving a discrete delimitation block in both ASAP and bPTP, without any overlap with *T. piperata*. The congruence across all independent lines of evidence (morphology, pigmentation pattern, COI distances and two modern species delimitation methods) provides strong support for the recognition of *T*. *personata* as a valid species. The increasing use of ASAP and bPTP in Neotropical ichthyology (e.g. Gales et al., 2022; Lima et al., 2025; Limeira Filho et al., 2024; Lopez et al., 2025; Martins et al., 2024) further reinforces the reliability and taxonomic relevance of these approaches.

The diversity of *Tympanopleura* consists primarily of Amazonian lineages (Walsh et al., 2015). The genus currently comprises seven species recognized as valid, some of which are widely distributed throughout the upper and middle Amazon River basin (e.g. *T*. *atronasus*, *T*. *longipinna* and *T*. *rondoni*). *T. piperata* is the only species in the genus found outside Amazon River basin, occurring in the Essequibo River drainage of Guyana, including the Potaro and Rupununi rivers (Fricke et al., 2025; Walsh et al., 2015).

The discovery of *T*. *personata* highlights the Purus River drainage as an understudied centre of diversity for Auchenipteridae. The occurrence of another putative undescribed species in the same basin (Ribeiro, personal observation; Hashimoto et al., 2020) indicates that the evolutionary history of *Tympanopleura* is more complex than previously recognized, likely shaped by hydrological isolation and habitat heterogeneity across western Amazonia. The congruence among morphological, molecular and species‐delimitation approaches not only supports the validity of *T. personata* but also underscores the importance of integrative taxonomic efforts for revealing hidden diversity. Considering the restricted distribution of *T. personata* and ongoing environmental alterations in the region, the formal description of the species provides an essential baseline for future ecological and conservation assessments.

### 4.1 Key to the species of *Tympanopleura*

**1**. Body with dark‐brown to black irregular spots scattered over most of body, usually heavily concentrated on head and upper half of body, or with a large dark blotch of melanophores on flanks above anal‐fin base, streaks in each caudal‐fin lobe and dark pigment on chin (pigmentation occasionally light or faded in preserved specimens) ................................................................................................................................. **2**

**1′**. Body with relatively uniform pigmentation, consisting of light to moderate stippling, darkest on top of head, dorsum and sides, lacking well‐defined spots or large blotches .................................................................................... **3**

**2**. Pectoral‐fin rays 7–9; total gill rakers on anterolateral margin of first arch usually 14–18, rarely greater (to 23); anal‐fin rays 23–30, usually 25–29; pigmentation of body consisting of dense patches of dusky specks on chin and on sides above anal fin; an elongate stripe in each caudal lobe, extending from base of hypural plate to midpoint of longest caudal rays or beyond; pectoral, pelvic and caudal fins often with dark marginal band ............................................................... *T*. *atronasus*

**2′**. Pectoral‐fin rays 10–13; total gill rakers on anterolateral margin of first arch 24–33; anal‐fin rays 28–37; pigmentation of body consisting of dark‐brown to black irregular spots extensively distributed on head, dorsum, sides of body and fins ............................ *T*. *rondoni*

**3**. Anal‐fin rays 31–42 ............................................................................. **4**

**3′**. Anal‐fin rays 23–30 ............................................................ *T*. *cryptica*

**4**. Dorsal profile of head moderately depressed, dorsal profile weakly convex from tip of snout to dorsal‐fin origin; pectoral‐fin spine short, 13.9%–18.8% *L*_S_, not reaching past pelvic‐fin origin; anal‐fin base long, 33.9%–42.4% *L*_*S*_; predorsal length 27.2%–36.1% *L*_*S*_ ........... **5**

**4′**. Dorsal profile of head greatly depressed anterior to eyes, acutely angled upwards from rear margin of fontanelle to dorsal origin, concave, especially in nuptial males; pectoral‐fin spine long, 19.1%–24.4% *L*_S_, often reaching to or past pelvic‐fin origin; anal‐fin base short, 26.6%–33.9% *L*_S_; predorsal length 35.1–44.0% *L*_S_ .................................................................................................................... *T*. *brevis*

**5**. Prominent transverse bar of dark melanophores at base of caudal fin (pigmentation occasionally inconspicuous); small body width at the pectoral‐fin origin, 13.0%–20.0% *L*_S_; great eye diameter 24.3%–35.7% *L*_H_ .............................................................................................................. **6**

**5′**. Scattered pigmentation at base of caudal fin, but not forming an inconspicuous or distinctive hourglass‐shaped transverse bar; great body width at the pectoral‐fin origin, 21.1–25.7 *L*_S_; small eye diameter 11.6%–18.5% *L*_H_ …........................................................................ *T*. *longipinna*

**6**. Surface of the head covered with diffuse minute brown specks consisting of individual melanophores, occasionally enlarged or coalesced and forming weak spots or slight mottling; short caudal peduncle length, 7.6%–10.5% *L*_S_; long distance between dorsal‐fin end and adipose‐fin origin, 45.9%–55.4% *L*_S_ …..........................................*T*. *piperata*

**6′**. Colouration of the head consisting of the combination of an intensely pigmented square‐shaped blotch on the supraoccipital and a dark‐brown blotch above each eye, shaped like a semicircle; long caudal peduncle length, 11.1%–13.8% *L*_S_; small distance between dorsal‐fin end and adipose‐fin origin 41.9%–45.6% *L*_S_ …........................................*T*. *personata*

### 4.2 Comparative material examined

All from South America: *A. lineatus*: **Brazil**: Amazonas: INPA 35931, 2 (68.8–75.4 mm *L*_S_, 1 c&s), Negro River. Pará: INPA 33975, 2 (113.5–125.0 mm *L*_S_, 1 dry skeleton), Tapajós River. UFOPA‐I 665, 2 (95.4–130.5 mm *L*_S_), Arapiuns River. *T. atronasus*: MCZ 27270, holotype (72.2 mm *L*_S_, male), exact locality unknown. **Bolivia**: Beni: FMNH 58138, 1 (94.4 mm *L*_S_), San Joaquin; FMNH 58139, 1 (69.2 mm *L*_S_), Guaporé River. **Brazil**: Acre: INPA 28507, 1 (84.1 mm *L*_S_), Purus River. Amapá: MZUSP 101875, 1 (120.9 mm *L*_S_), Jarí River. Amazonas: INPA 13392, 1 (103.7 mm *L*_S_), Solimões River; INPA 16598, 1 (74.3 mm *L*_S_), Amazon River; INPA 17176, 1 (91.1 mm *L*_S_), Purus River. Pará: MZUSP 7861, 1 (83.1 mm *L*_S_), Nhamundá River; MZUSP 9285, 1 (109.0 mm *L*_S_), Itapiranga. Rondônia: INPA 10955, 2 (93.3–101.1 mm *L*_S_), Madeira River; INPA 35928, 3 (91.9–107.9 mm *L*_S_), Guaporé River; INPA 35929, 3 (123.3–146.8 mm *L*_S_), Guaporé River. **Peru**: Loreto: ANSP 178257, 2 (74.4–105.0 mm *L*_S_), Amazon River; ANSP 178307, 4 (46.2–74.7 mm *L*_S_), Amazon River. Ucayali: FMNH 93488, 3 (100.8–109.9 mm *L*_S_), Yarinococha; MZUSP 113998, 1 (75.9 mm *L*_S_), Ucayali River; USNM 261396, 2 (72.0–85.5 mm *L*_S_), Ucayali River. *T. brevis*. **Brazil**: Amazonas: NMW 47801, 2, syntypes (95.4–104.0 mm *L*_S_), Amazon River at Coary; ANSP 193972, 1 (56.2 mm *L*_S_), Negro River; INPA 10311, 16 (40.1–77.3 mm *L*_S_, 1 c&s), Negro River; INPA 18528, 17 (44.2–60.8 mm *L*_S_, 2 c&s), Purus River. **Peru**: Loreto: ANSP 182476, 1 (85.0 mm *L*_S_), Nanay River. *T. cryptica*. **Brazil**: Amazonas: INPA 12609, paratype (67.5 mm *L*_S_), Ilha da Marchantaria; INPA 18986, paratype (66.9 mm *L*_S_), Tefé, Japurá River; INPA 25112, paratype (78.2 mm *L*_S_), Madeira River; INPA 35926, paratypes (54.1–72.0 mm *L*_S_, 1 c&s), Purus River. **Peru**: Loreto: MUSM 47102, holotype (84.9 mm *L*_S_, prenuptial male), Solimões River; ANSP 139065, paratype (59.0 mm *L*_S_), Nanay River. **Brazil**: Rondônia: MZUSP 114000, holotype (73.0 mm *L*_S_, female), Madeira River. *T. longipinna*. **Bolivia**: Beni: AMNH 56069, paratype (63.2 mm *L*_S_), Mamoré River. **Brazil**: Amazonas: ANSP 193974, 5, paratypes (52.1–65.9 mm *L*_S_), Japurá River; ANSP 194004, 18, paratypes (57.5–75.5 mm *L*_S_), Madeira River; MZUSP 114002, 2, paratypes (63.6–66.0 mm *L*_S_), mouth of Ituxi River. Rondônia: MZUSP 34418, 4, paratypes (68.4–75.7 mm *L*_S_), Madeira River. **Peru**: Loreto: ANSP 178310, 11 paratypes (43.1–76.9 mm *L*_S_), Amazon River; USNM 124918, 3, paratype (64.3–68.2 mm *L*_S_). Shansho Cano: MZUSP 25972, 4, paratypes (41.5–65.3 mm *L*_S_), Ucayali River. *T. piperata*. **Brazil**: Amazonas: ANSP 194019, 3 (36.9–42.5 mm *L*_S_), Jutaí River; ANSP 194022, 5 (43.2–47.5 mm *L*_S_), Negro River; INPA 10263, 1 (41.4 mm *L*_S_), Negro River; INPA 12603, 11 (24.4–31.6 mm *L*_S_), Jaú River; MZUSP 56200, 3 (unmeasured), Jutaí River. Rondônia: UNIR 1589, 20 (41.1–54.0 mm *L*_S_), Madeira River. **Guyana**: FMNH 53243, holotype, 1 (47.3 mm *L*_S_, male), Essequibo River at Crab Falls; FMNH 53244 paratype, 1 (41.7 mm *L*_S_), same collection data as holotype. CAS 58382, 3, paratypes (44.6–47.5 mm *L*_S_), same collection data as holotype. MCZ 30189, paratype (46.4 mm *L*_S_, female), same collection data as holotype. BMNH 1911.10.31.102, paratype (44.8 mm *L*_S_, male), same collection data as holotype; ANSP 175838, 1 (46.4 mm *L*_S_), Essequibo River at Essequibo; ANSP 179676, 2 (33.7–38.4 mm *L*_S_), Rupununi River. **Peru**: Loreto: ANSP 191444, 4 (42.5–37.4 mm *L*_S_), Nanay River; ANSP 191445, 5 (32.5–35.0 mm *L*_S_), Nanay River; MZUSP 115006, 2 (38.1–38.4 mm *L*_S_), Nanay River. *T. rondoni*. **Bolivia**: Beni: MZUSP 27805, 3 (79.5–110.8 mm *L*_S_), Laguna San José; MZUSP 114008, 2 (51.2–55.7 mm *L*_S_), Trinidad; MZUSP 37880, 1 (124.4 mm *L*_S_), Beem Stream, Humaitá. **Brazil**: Amazonas: MNRJ 962A, lectotype (160.4 mm *L*_S_), Negro River; MNRJ 962, paralectotype (134.4 mm *L*_S_), Negro River; ANSP 193989, 2 (63.3–77.0 mm *L*_S_), Purus River; ANSP 194016, 5 (99.3–124.7 mm *L*_S_), Solimões River; INPA 18961, 1 (115.7 mm *L*_S_), Japurá River; INPA 22139, 7 (113.8–156.0 mm *L*_S_), Solimões River; INPA 26537, 1 (115.6 mm *L*_S_), Negro River; MCP 29877, 1 (88.0 mm *L*_S_), Solimões River; MZUSP 56649, 1 (73.1 mm *L*_S_), Purus River; MZUSP 56656, 1 (75.6 mm *L*_S_), Amazon River. Pará: INPA 22722, 2 (115.0–116.0 mm *L*_S_), Amazon River. Rondônia: INPA 21720, 1 (117.7 mm *L*_S_), Madeira River; UFRO‐I 682, 2 (53.3–95.6 mm *L*_S_), Madeira River.

## AUTHOR CONTRIBUTIONS

Cárlison Silva‐Oliveira and Valdenor Magalhães collected the specimens. Frank Raynner V. Ribeiro, Lúcia H. Rapp Py‐Daniel and Cárlison Silva‐Oliveira contributed to the original idea, development of the study and writing of the manuscript. Valdenor Magalhães and Lucas Gama generated molecular data. All authors contributed to the development of the study, writing the manuscript and approving the final version.

## FUNDING INFORMATION

Frank Raynner V. Ribeiro and Cárlison S. Oliveira are funded by the Conselho Nacional de Desenvolvimento Científico e Tecnológico (CNPq process 310480/2022‐1 and 317781/2021‐9, respectively) and Fundação Amazônia de Amparo a Estudos e Pesquisas do Pará (Fapespa process 2023/158262). Frank Raynner V. Ribeiro was partly supported by Instituto de Conservação Ambiental The Nature Conservancy do Brasil (Águas Tapajós Project, Cooperation BR FY23 264).

## APPENDIX A GENBANK ACCESSION NUMBER FOR THE COI SEQUENCES OF THE TYMPANOPLEURA SPECIES USED IN THIS STUDY

**Table:**

Species	Voucher	Country	Basin	GenBank number	Reference
*Tympanopleura atronasus*	UFRO‐I 017923, UFRO‐I 013955	Brazil	Madeira River	MN460972, MN460971, MN460975, MN460978	Hashimoto et al. (2020)
*Tympanopleura brevis*	INPA 44373	Brazil	Purus River	MN460981, MN460982	Hashimoto et al. (2020)
*Tympanopleura longipinna*	INPA 44374	Brazil	Purus River	MN460986, MN460983	Hashimoto et al. (2020)
*T. longipinna*	ANSP 178310	Peru	Amazon River	MT712807, MT712808	Sabaj & Arce (2021)
*Tympanopleura personata*	INPA 61048	Brazil	Ituxi River	PV261062, PV261063	This study
*Tympanopleura piperata*	INPA 41946	Brazil	Purus River	MN460987, MN460988	Hashimoto et al. (2020)
*T. piperata*	ROM 86042	Guyana	Essequibo River	MT712809	Sabaj & Arce (2021)
*Tympanopleura rondoni*	No catalogue	Brazil	Madeira River	MN461000, MN461001, MN460997	Hashimoto et al. (2020)
*T. rondoni*	INPA 41761	Brazil	Purus River	MN460996	Hashimoto et al. (2020)
*Tympanopleura* sp.	No catalogue	Brazil	Madeira River	MN461003, MN461004	Hashimoto et al. (2020)
*Tympanopleura* sp.	INPA 46728	Brazil	Solimões River	MN461002	Hashimoto et al. (2020)

Abbreviations: COI, cytochrome oxidase, subunit I; INPA, Instituto Nacional de Pesquisas da Amazônia.
